# A Quaternary Ammonium-Modified Resin for Selective Perchlorate Removal from Fireworks Wastewater

**DOI:** 10.3390/polym18050553

**Published:** 2026-02-25

**Authors:** Fei He, Jiacheng Li, Zhipeng Pei, Yuhao Zhao, Yiping Li

**Affiliations:** 1 State Key Laboratory of Water Cycle and Water Security in River Basin, College of Environment, Hohai University, Nanjing 210098, China; hefei@nies.org (F.H.); 241605010016@hhu.edu.cn (J.L.); 2 Nanjing Institute of Environmental Sciences, Ministry of Ecology and Environment, Nanjing 210042, China; peizhipeng@nies.org (Z.P.); zhaoyuhao.cn@gmail.com (Y.Z.)

**Keywords:** perchlorate, resin, quaternary ammonium

## Abstract

Perchlorate (ClO_4_^−^) is difficult to remove efficiently using conventional treatment technologies, such as coagulation and reverse osmosis, due to its high water solubility and exceptional chemical stability. Quaternary ammonium resins have emerged as cost-effective and efficient materials for ClO_4_^−^ removal; therefore, the development of high-performance quaternary ammonium resins is critical for improving ClO_4_^−^ remediation. In this study, a novel resin (PS-QA) was synthesized by aminating poly (vinylbenzyl chloride) with *N*,*N*-dimethylethanolamine, and its adsorption performance was systematically compared with that of three internationally recognized commercial ClO_4_^−^ removal resins. Although all four resins exhibited spherical morphologies, the polystyrene backbone exhibited strong hydrophobicity, and the functional group –[R-N^+^(CH_3_)_2_(C_2_H_4_OH)]Cl^−^ possesses good electrophilicity, thereby conferring excellent selectivity toward ClO_4_^−^. PS-QA exhibited a specific surface area of 19.94 m^2^/g, an average pore diameter of 32 nm, and a pore volume of 0.157 cm^3^/g, indicating comparable adsorption performance relative to the commercial counterparts. Its high thermal stability was further demonstrated through thermogravimetric analysis. Adsorption equilibrium was reached within 60 min, and the kinetic performance of PS-QA was comparable to that of the commercial resins. Isotherm analysis showed that ClO_4_^−^ adsorption conformed to the Freundlich model, suggesting a coupled physical–chemical adsorption mechanism. Moreover, PS-QA exhibited both strong resistance to interference in complex water matrices and excellent reusability. After three adsorption–desorption cycles, more than 80% of the adsorption sites remained active. Notably, PS-QA also demonstrated outstanding performance in pilot-scale applications.

## 1. Introduction

Perchlorate (ClO_4_^−^) is widely used as a strong oxidizing agent in applications such as fireworks and pyrotechnic manufacturing [1,2]. China has a long history of fireworks production, with numerous enterprises engaged in this industry [3,4,5]. However, due to the extremely high water solubility and exceptional chemical stability of ClO_4_^−^, large volumes of ClO_4_^−^-containing wastewater are generated annually [6,7]. When this wastewater is discharged without adequate treatment, ClO_4_^−^ contaminants may enter the human body through drinking water, accumulate over time, disrupt thyroid function, and induce a range of adverse health effects [8,9,10]. With accelerating industrialization, ClO_4_^−^ contamination has gradually evolved from a localized concern into a global environmental challenge, posing serious threats to drinking water safety and ecosystem health [11].

Conventionally, ClO_4_^−^ can be removed through conventional treatment methods such as coagulation, precipitation, filtering, and disinfection [12,13,14,15]. However, these technologies are often associated with high operational costs, limited regenerability, and risks of secondary pollution [16,17]. In comparison, resin adsorption technologies offer greater economic feasibility and operational simplicity, thereby exhibiting substantial application potential [18,19]. Nevertheless, unmodified resins generally suffer from low adsorption capacities, slow adsorption kinetics, and insufficient selectivity toward ClO_4_^−^, underscoring the urgent need for the development of advanced modified resin materials.

Functional group modification is widely recognized as an effective strategy for enhancing the overall performance of resins [20]. Appropriately designed functional groups can promote efficient ClO_4_^−^ adsorption through the combined effects of physical and chemical interactions [21]. For example, Li et al. [22] synthesized quaternary ammonium–functionalized resins with exceptional selectivity for ClO_4_^−^, while Liu et al. [23] developed –[R-N^+^(CH_3_)_3_]Cl^−^ functionalized resins that exhibit significantly accelerated adsorption kinetics. These studies collectively demonstrate that the introduction of quaternary ammonium groups via substitution reactions can markedly improve the ClO_4_^−^ adsorption performance of a resin. In addition to functional groups, the matrix, pore size, and specific surface area of a resin are also critical determinants of its ClO_4_^−^ adsorption performance [24]. Because ClO_4_^−^ possesses relatively low hydration energy compared with other anions, hydrophobic polystyrene-based resins tend to exhibit strong affinity and superior selectivity for ClO_4_^−^ in complex water matrices containing NO_3_^−^, SO_4_^2−^, and Cl^−^ [25,26,27,28]. Larger pore sizes facilitate ClO_4_^−^ transport, while higher specific surface areas provide a greater number of available adsorption sites. Therefore, resins should be engineered with broad pore size distributions and large surface areas to achieve rapid adsorption kinetics and high ClO_4_^−^ removal efficiency.

In this work, poly vinylbenzyl chloride (PVBC) was employed as a polystyrene-based resin backbone, and *N*,*N*-dimethylethanolamine was used as the amination agent to synthesize a novel resin bearing –[R-N^+^(CH_3_)_2_(C_2_H_4_OH)]Cl^−^ functional groups. The adsorption performance of the resulting PS-QA resin was systematically evaluated and compared with that of three high-performance commercial ClO_4_^−^-removal resins (BAX01, BDX01, and PW521). The morphology, functional groups, pore structure, and specific surface area of the resins were characterized by scanning electron microscopy (SEM), Fourier transform infrared spectroscopy (FTIR), and Brunauer–Emmett–Teller (BET) analyses. PS-QA exhibited highly electrophilic functional groups favorable for efficient ClO_4_^−^ adsorption with a 95% confidence interval, the largest average pore diameter (32 nm), and specific surface area (19.94 m^2^/g) among the four resins. Thermogravimetric analysis further confirmed its excellent thermal stability. Comparative analyses of adsorption kinetics and isotherms demonstrated that PS-QA achieved rapid ClO_4_^−^ uptake and high adsorption capacity. Moreover, selectivity experiments revealed that PS-QA maintained strong selectivity toward ClO_4_^−^ even in the presence of competing anions (NO_3_^−^, SO_4_^2−^, and Cl^−^). Furthermore, regeneration experiments showed that PS-QA retained approximately 80% of its adsorption capacity after three adsorption–desorption cycles. Finally, PS-QA demonstrated outstanding performance when applied to actual fireworks wastewater, highlighting its strong potential for practical applications.

## 2. Materials and Methods

### 2.1. Chemicals and Resin Materials

All chemicals used in this study, including sodium perchlorate monohydrate (NaClO_4_⋅H_2_O), potassium nitrate (KNO_3_), potassium chloride (KCl), sodium sulfate (Na_2_SO_4_), humic acid (HA), hydrochloric acid (HCl), and sodium hydroxide (NaOH), were purchased from Sinopharm Chemical Reagent Co., Ltd. (Shanghai, China). The three commercial resins were supplied by Jiangsu Jinkai Resin Chemical Co., Ltd. (Yancheng, China). The synthesized PS-QA resin possessed a polystyrene backbone and was synthesized through the amination of chloromethyl polystyrene beads, which were also purchased from Jiangsu Jinkai Resin Chemical Co., Ltd. (China). *N*,*N*-dimethylethanolamine was used as the amination agent. The detailed synthesis procedure is provided in Supplementary Text S1 and Figure S1. All other chemicals used were of analytical grade and were obtained from Sinopharm Chemical Reagent Co., Ltd. (China).

### 2.2. Resin Characterization

The specific surface area and pore structure of PS-QA and the three commercial resins were determined by nitrogen adsorption–desorption measurements at 77 K using an automatic specific surface area and pore size distribution analyzer (TriStar II Plus 3030, Micromeritics, Norcross, GA, USA). Surface functional groups were identified by FTIR analysis using KBr pellets over the wavenumber range of 4000–1000 cm^−1^ (Nicolet iS20, Thermo Fisher Scientific, Waltham, MA, USA). The morphological characteristics of the resins were examined by SEM (GeminiSEM 360, ZEISS, Jena, Germany). The thermal stability of the resins was evaluated by thermogravimetric analysis (TGA 209 F1, NETZSCH, Selb, Germany). The concentration of ClO_4_^−^ in solution was determined using an ion chromatograph (Dionex ICS-600, Thermo Fisher Scientific, USA), and the solution pH was measured with a digital laboratory pH meter (PHS-25, Yantai Stark Instrument, Yantai, China).

### 2.3. Density Functional Theory (DFT) Calculations

Conceptual DFT calculations were performed using the Gaussian 09 package. Geometry optimization was conducted using the B3LYP functional in combination with the 6–31 G basis set. The wavefunction of PS-QA was analyzed using Multiwfn_3.8 software, and the DFT results were visualized accordingly [29]. The molecular model and electronic features of the resin were displayed using Visual Molecular Dynamics (VMD).

### 2.4. Adsorption Kinetics Experiments

In each adsorption kinetics experiment, 200 mL of a 200 mg/L ClO_4_^−^ solution was contacted with 0.2 g of a resin in a thermostatic oscillating water bath at 295, 305, or 315 K, and the shaking speed was 120 rpm. The mixture was agitated using a thermostatic orbital shaker operated at 120 rpm. At each predetermined time interval (15 min, 30 min, 1 h, 2 h, 4 h, 8 h, 20 h, and 24 h), a 2 mL aliquot was withdrawn from the solution for analysis. For each temperature, adsorption experiments were conducted in triplicate. The kinetic data were fitted using the pseudo-first-order, pseudo-second-order, and intraparticle diffusion models (Text S2) [30]. ClO_4_^−^ uptake was calculated using Equation (1):

(1) $$q_{t}=V\cdot (C_{0}-C_{t})/W$$

where *V* (L) and *W* (g) represent the solution volume and resin mass, respectively, while $C_{0}$ (mg/L) and $C_{t}$ (mg/L) denote the ClO_4_^−^ concentrations at *t* = 0 and at time *t* (min), respectively.

### 2.5. Adsorption Isotherm and Equilibrium Experiments

Batch adsorption isotherm experiments were conducted using ClO_4_^−^ solutions with initial concentrations ranging from 50 to 500 mg/L. In each experiment, 100 mL of a ClO_4_^−^ solution was contacted with 0.1 g of a resin in a thermostatic oscillating water bath at 295, 305, or 315 K, and the shaking speed was 120 rpm. During the 48 h equilibration period, the ClO_4_^−^ solution was agitated at 120 rpm, with its pH maintained constant. After equilibrium was reached, the uptake of ClO_4_^−^ by the resin was calculated. The adsorption isotherm behavior was then described using the Langmuir and the Freundlich isotherm models (Text S3) [31,32].

### 2.6. Effects of pH, Salinity, and Dissolved Organic Matter (DOM) on ClO_4_^−^ Adsorption

For each fixed pH condition, the initial pH of a 200 mg/L ClO_4_^−^ solution (200 mL) was adjusted to 2, 4, 7, 10, or 12 using a 1 M HCl or NaOH solution. Subsequently, 0.25 g of a resin was added, and the suspension was shaken at 298 K and 120 rpm for 48 h. Similarly, the effects of salinity on ClO_4_^−^ uptake by the resin were investigated under different concentrations of NO_3_^−^, SO_4_^2−^, or Cl^−^. For each of these competing anions, five concentrations were employed: 0.5, 2.0, 5.0, 10.0, and 20.0 mM. The influence of DOM on ClO_4_^−^ uptake was evaluated using different HA concentrations (10, 20, 50, 80, and 100 mg/L). The procedures used to assess the effects of pH, salinity, and DOM were consistent with those applied in the adsorption equilibrium and isotherm experiments. For each experimental condition, adsorption experiments were conducted in triplicate.

### 2.7. Reusability Experiments

PS-QA was saturated with ClO_4_^−^ by mixing 0.8 g of the resin with 200 mL of a 2000 mg/L ClO_4_^−^ solution, followed by shaking at 298 K and 120 rpm for 2 days. Desorption kinetics were then evaluated by vortexing a mixture containing 30 mL of regenerant solution and 0.1 g of the saturated resin at 120 rpm and 295 K.

In the reusability experiments, NaCl solutions with different concentrations (5, 10, and 15%) were used as the regenerants. The adsorption–desorption process was repeated three times to assess the reusability of the resin, with each desorption cycle lasting 2 h. After desorption, the regenerated resin was washed twice with deionized water and then prepared for the subsequent adsorption cycle. The regeneration efficiency is calculated as the ratio of the resin’s equilibrium adsorption capacity after the *n*-th cycle to that of the first cycle.

### 2.8. DFT

Density functional theory (DFT) calculations were performed to investigate the structural and electronic properties of the target systems. All geometries were fully optimized using the B3LYP functional with the 6-31 G (d, p) basis set, which includes polarization functions on both heavy atoms and hydrogen atoms. Monomeric units were adopted as the computational models in this study. The total energy tolerance was set to 10^−5^ eV, and the force tolerance was 0.02 eV/Å. The SMD (Solvation Model based on Density) model was used to simulate the aqueous environment, with water as the solvent. Electronic property analysis: The electrostatic potential was calculated using the Merz–Kollman scheme, and the electron density distribution was analyzed via Multiwfn_3.8 software.

## 3. Results and Discussion

### 3.1. Structural and Physicochemical Characterization of Resins

#### 3.1.1. Morphology and Surface Feature Analysis

**Figure 1:**
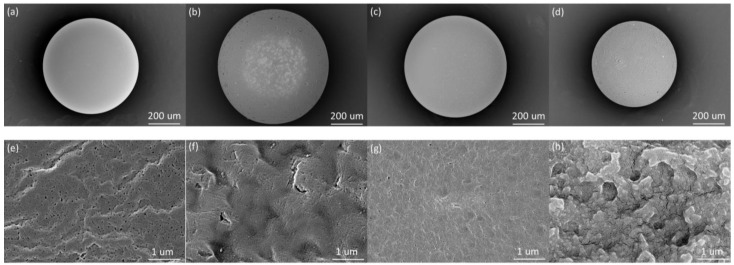
SEM of PS-QA (**a**,**e**), BAX01 (**b**,**f**), BDX01 (**c**,**g**), and PW521 (**d**,**h**).

PS-QA exhibited a yellow coloration, whereas the three commercial resins appeared white or pale yellow (Figure S2). The average particle diameters of PS-QA, BAX01, BDX01, and PW521 were approximately 600, 700, 650, and 550 μm, respectively. Because smaller particle sizes are generally associated with larger specific surface areas, the particle size of PS-QA falls within the mid-to-lower range relative to those of the commercial resins. The morphologies of the four resins were further characterized by SEM (Figure 1). All resins displayed spherical geometries. The surface of PS-QA was smooth and free of obvious defects, whereas pronounced grooves and scratches were observed on BAX01 and PW521 (Figure 1b,d), which were likely introduced during pretreatment involving magnetic stirring. This observation suggests that PS-QA possesses superior physical robustness compared with the commercial resins. At higher magnification, PS-QA and BDX01 exhibited similar surface features (Figure 1e,g); however, PS-QA displayed a larger surface pore size, which is more favorable for ClO_4_^−^ adsorption.

#### 3.1.2. Functional Group Analysis

**Figure 2:**
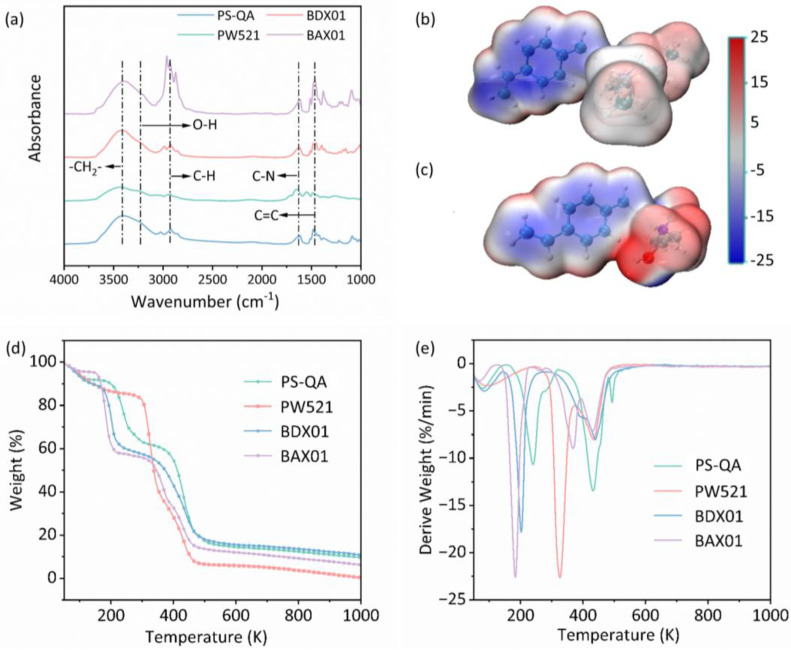
(**a**) The FTIR spectra of PS-QA, BAX01, BDX01, and PW521. (**b**) Surface electrostatic potential in (**b**) BDX01 and (**c**) PS-QA. TG (**d**) and DTG (**e**) of PS-QA, BAX01, BDX01, and PW521.

To further elucidate the functional groups present in the four resins, the FTIR spectra of the four materials were recorded over the wavenumber range of 1000–4000 cm^−1^ (Figure 2a). The absorption band near 3400 cm^−1^ was attributed to the stretching vibrations of the –CH_2_– groups in those polymeric organic materials [23]. The broad band around 3300 cm^−1^ corresponded to O–H stretching vibrations, while the broad absorption near 3200 cm^−1^ was attributed to carboxylic O–H groups [33]. The intense absorption band around 3000 cm^−1^ corresponded to C–H stretching vibrations [34], the intensity and profile of which reflected the bonding environment and distribution of organic functional groups in the four resins. The band at approximately 1600 cm^−1^ was attributed to C–N stretching vibrations, whereas the band near 1450 cm^−1^ was assigned to the skeletal vibrations of the aromatic ring [35].

Collectively, these spectral features indicate that PS-QA, BDX01, and BAX01 possess polystyrene backbones, while PW521 is based on a polyacrylic matrix, explaining the presence of –CH_2_– stretching vibrations in all four resins. Owing to the carboxylic functionality of polyacrylic acid, PW521 exhibits a broad O–H absorption band near 3200 cm^−1^. In contrast, the other three resins contain aromatic rings, giving rise to characteristic skeletal vibrations near 1450 cm^−1^. PS-QA contains C_2_H_4_OH moieties, resulting in a pronounced broad band around 3300 cm^−1^. All four resins bear quaternary ammonium functionalities, leading to strong C–H stretching vibrations near 3000 cm^−1^. Notably, BAX01, functionalized with –[R-N^+^(CH_2_CH_2_CH_3_)_3_]Cl^−^, contains a greater number of C–H bonds and therefore exhibits a markedly stronger absorption intensity. The presence of C–N stretching bands near 1600 cm^−1^ further confirms the quaternary ammonium nature of all resins. Based on these spectral analyses, together with manufacturer-provided information, BDX01 and BAX01 are confirmed to be polystyrene-based resins, whereas PW521 is polyacrylic-based; BDX01 and PW521 contain –[R-N^+^(CH_3_)_3_]Cl^−^ functionalities, while BAX01 contains –[R-N^+^(CH_2_CH_2_CH_3_)_3_]Cl^−^ groups. PS-QA likewise features a polystyrene backbone functionalized with –[R-N^+^(CH_3_)_2_(C_2_H_4_OH)]Cl^−^ groups.

#### 3.1.3. Electronic Structure and Electrostatic Potential Analysis

Following identification of resin backbones and functional groups, the molecular models of the four resins were constructed using GaussView, and their electrostatic potentials were calculated with Gaussian to elucidate charge distributions and reactive sites. The resulting electrostatic potential maps were visualized using VMD and are presented in Figure 2b,c and Figure S3, in which red and blue regions represent positive and negative electrostatic potentials, respectively. Notably, PS-QA exhibits a higher positive ESP value (+39.94 kcal/mol) compared to BDX01 (+12.42 kcal/mol) and BAX01 (+13.16 kcal/mol). The functional groups of PS-QA exhibited pronounced electron-deficient characteristics (Figure 2c), which favor strong electrostatic interactions with negatively charged ClO_4_^−^. In contrast, the functional groups of BDX01 (Figure 2c) and BAX01 were predominantly positively charged or near neutral, resulting in comparatively weaker affinity toward ClO_4_^−^. Although PW521 also displayed electron-deficient functional groups, its polyacrylic backbone is substantially more hydrophilic than the polystyrene backbone of PS-QA, leading to strong affinity toward highly hydrated anions such as sulfate (1103 kJ/mol), chloride (371 kJ/mol), and nitrate (314 kJ/mol). Consequently, PW521 preferentially adsorbs these competing anions, which occupy the adsorption sites of the resin and thereby hinder ClO_4_^−^ uptake. Because ClO_4_^−^ has a significantly lower hydration energy (205 kJ/mol) compared to most common anions, hydrophobic polystyrene-based resins inherently exhibit higher affinity for ClO_4_^−^. By combining a hydrophobic polystyrene backbone with electron-deficient functional groups, PS-QA demonstrates a highly favorable electronic structure for selective ClO_4_^−^ removal from complex water matrices. The polystyrene backbone provides a predominantly hydrophobic surface, which is beneficial for the adsorption of perchlorate (ClO_4_^−^) owing to its low hydration energy. The hydroxyl groups present localized sites capable of forming hydrogen bonds with water molecules and adjusting the arrangement of interfacial water, but they do not change the overall hydrophobic nature of the adsorbent. Meanwhile, the electron-withdrawing effect of the hydroxyethyl groups enhances the electrostatic interaction between the adsorbent and perchlorate anions, thereby improving the adsorption selectivity.

#### 3.1.4. Thermal Stability Analysis

Thermogravimetric analysis was conducted to assess the thermal and physical stability of the four resins. All resins exhibited mass loss that occurred primarily in three distinct stages (Figure 2d,e). At temperatures below 100 °C, the observed weight loss was attributed to the release of residual H_2_O, CO_2_, and entrapped solvents. The second stage, occurring between 200 and 250 °C, was mainly associated with the decomposition of functional groups [36], while degradation of the polymer backbone occurred at approximately 400 °C [37]. Among the four resins, BAX01 exhibited the most pronounced mass loss near 200 °C, indicating a relatively higher content of functional groups. Owing to its distinct polyacrylic backbone, PW521 displayed a markedly different thermogravimetric profile compared with the polystyrene-based resins (PS-QA, BDX01, and BAX01). Among these polystyrene-based resins, PS-QA exhibited the highest thermal resistance, with functional group decomposition initiating at approximately 250 °C. Overall, PS-QA demonstrated thermal stability comparable to that of the commercial resins.

#### 3.1.5. Surface Area and Pore Structure Analysis

Among the four resins, PS-QA exhibited the largest specific surface area, average pore diameter, and pore volume (Table 1), surpassing those of the commercial resins. These characteristics indicate an appropriate degree of crosslinking and a more developed pore structure, which together provide abundant adsorption sites for ClO_4_^−^. Notably, as indicated by FTIR analysis, the –[R-N^+^(CH_2_CH_2_CH_3_)_3_]Cl^−^ functional groups in BAX01 possessed long alkyl chains and occurred in relatively high abundance. Moreover, an excessive density of functional groups may increase the degree of crosslinking, potentially decreasing the number of pores and the accessible surface area, thereby limiting ClO_4_^−^ adsorption capacity.

### 3.2. Adsorption Kinetics and Temperature Effects

Following characterization of the physicochemical properties of the four resins, their ClO_4_^−^ adsorption performance was evaluated. The effect of initial solution pH was first examined (Figure S4). Only slight decreases in ClO_4_^−^ adsorption were observed under either acidic or alkaline conditions, indicating that PS-QA is largely insensitive to pH variations. The three commercial resins exhibited similar pH tolerance. In addition, the influence of pH on PS-QA was further confirmed by zeta potential characterization (Figure S5). Perchlorate uptake by strong base anion exchange resins may be nearly independent of solution pH, because their zeta potentials remain stable under both acidic and alkaline conditions [38]. Given that the pH of actual fireworks wastewater ranged from 6.8 to 8.0 (Table S3) and that PS-QA exhibited optimal performance at pH 7, all subsequent experiments were conducted under neutral pH conditions.

**Figure 3:**
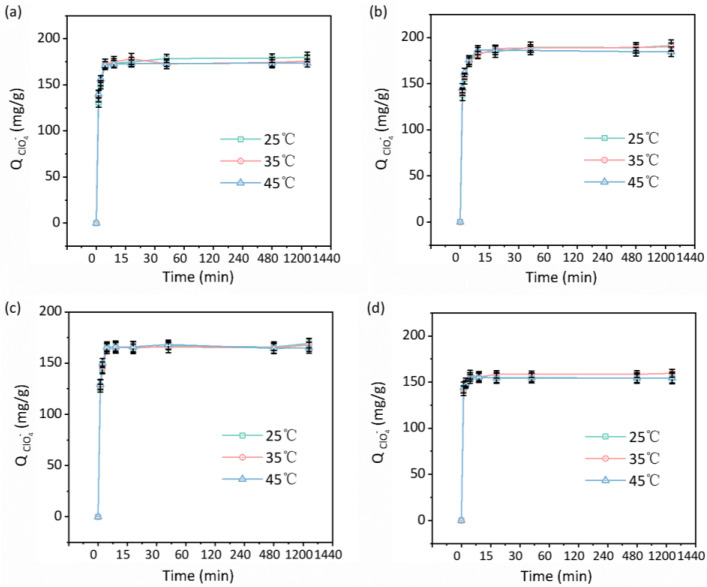
Adsorption kinetics of perchlorate on four representative resins at different temperatures: (**a**) PS-QA, (**b**) BDX01, (**c**) BAX01, and (**d**) PW521.

For all four resins, within the first 60 min, increases in temperature led to slight improvements in ClO_4_^−^ adsorption capacity (Figure 3), suggesting enhanced ClO_4_^−^ diffusion at elevated temperatures. However, temperature exerted a minimal influence on the equilibrium adsorption capacities. Both pseudo-first-order and pseudo-second-order kinetic models were applied to the experimental data, and the corresponding rate constants (K_1_ and K_2_) rose moderately with increasing temperature (Table S1). Notably, PS-QA exhibited K_1_ and K_2_ values slightly higher than those of BDX01 and BAX01, demonstrating that its adsorption kinetics were comparable to or exceeded those of the commercial resins. PW521 displayed the highest rate constants, likely due to its larger surface pore size. The correlation coefficients (R^2^ values) for both kinetic models exceeded 0.98 for all resins, indicating that the adsorption behavior is complicated.

### 3.3. Adsorption Isotherms and Thermodynamic Characteristics of ClO_4_^−^ Uptake

**Figure 4:**
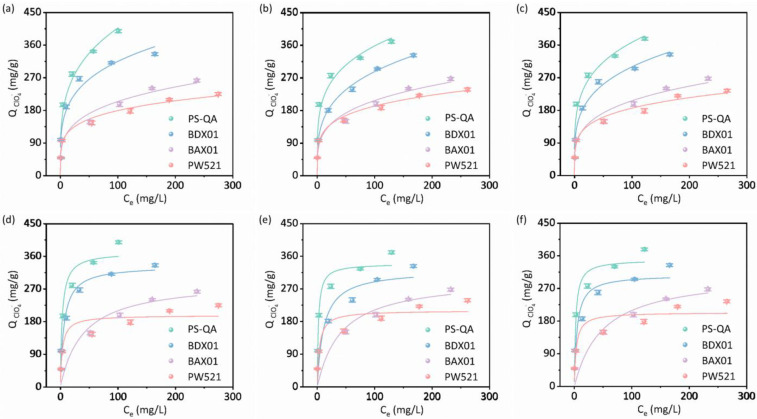
Effect of initial concentration of ClO_4_^−^ and fitted plots of Freundlich adsorption isothermal model on PS-QA, BAX01, BDX01, and PW521 at different temperatures: (**a**) 295 K, (**b**) 305 K, and (**c**) 315 K. Effect of initial concentration of ClO_4_^−^ and fitted plots of Langmuir adsorption isothermal model on PS-QA, BAX01, BDX01 and PW521 at different temperatures: (**d**) 295 K, (**e**) 305 K, and (**f**) 315 K.

Adsorption isotherm analysis (Figure 4) revealed that PS-QA exhibited a substantially higher adsorption capacity compared to the commercial resins, which is consistent with its larger surface area and pore volume. A slight decrease in adsorption capacity was observed with increasing temperature, likely due to enhanced ClO_4_^−^ desorption at elevated temperatures. Both the Langmuir and the Freundlich isotherm models were applied to describe the adsorption process, with the Freundlich model providing a significantly better fit to the experimental data (Table S2). Based on the Langmuir model simulations in Table S2, the resin PS-QA exhibits a higher adsorption capacity for perchlorate (371.3 mg/g) at room temperature compared to the commercial resins BDX01 (334.1 mg/g) and BAX01 (293.5 mg/g). Additionally, according to the Freundlich model, the adsorption capacity constant K_F_ for PS-QA at room temperature is 116.3, which is greater than those of the commercial resins BDX01 (101.6) and BAX01 (62.0). The superior fit of the Freundlich model suggests the presence of heterogeneous surface energies and multilayer adsorption, as well as the coexistence of multiple adsorption mechanisms [39,40], thereby further supporting the involvement of both physical and chemical interactions in ClO_4_^−^ uptake.

### 3.4. Selectivity Toward ClO_4_^−^ in the Presence of Competing Anions

**Figure 5:**
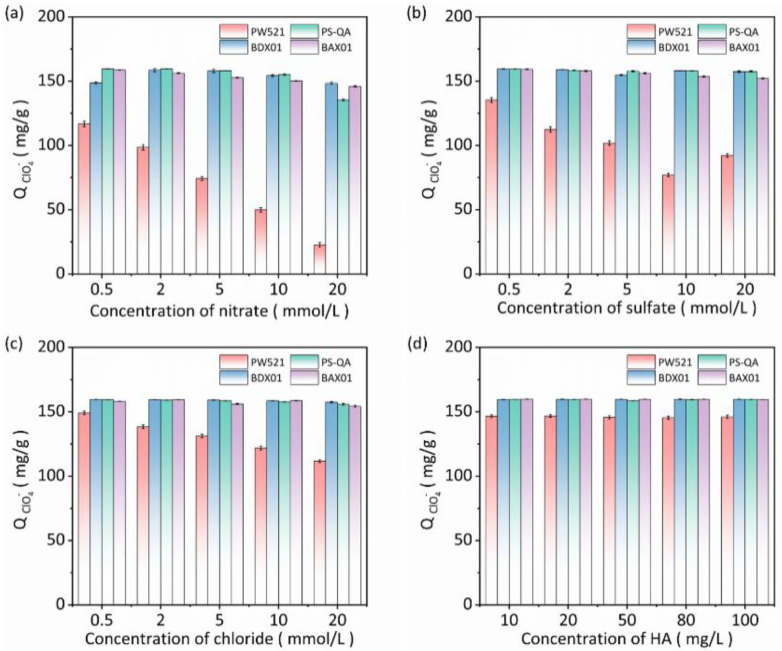
Perchlorate uptake by four resins at different salinity levels: (**a**) binary ClO_4_^−^/NO_3_^−^ system; (**b**) binary ClO_4_^−^/SO_4_^2−^ system; (**c**) binary ClO_4_^−^/Cl^−^ system. (**d**) Effect of HA concentration on perchlorate.

The presence of competing anions (Cl^−^, SO_4_^2−^, and NO_3_^−^) exerted minimal influence on ClO_4_^−^ adsorption by the polystyrene-based resins (Figure 5a–c), confirming their high selectivity toward ClO_4_^−^. This behavior can be attributed to the significantly lower hydration energy of ClO_4_^−^ (205 kJ/mol) compared with those of the competing anions, which facilitates its interaction with the hydrophobic surfaces of the resins [41]. In contrast, PW521, owing to its polyacrylic backbone, exhibited a stronger affinity for all anions, resulting in competitive occupation of adsorption sites and a corresponding decrease in ClO_4_^−^ uptake as the concentrations of the competing anions increased. When the concentration of HA was increased from 10 to 100 mg/L (Figure 5d), only slight changes in ClO_4_^−^ adsorption were observed for all resins, indicating that pore-blocking effects induced by HA were negligible. Overall, PS-QA demonstrated strong resistance to interference in complex water matrices, with performance comparable to that of the commercial resins. To illustrate the adsorption selectivity of the resins, the distribution coefficients (K_d_) were calculated under different concentrations of competing anions (NO_3_^−^, Cl^−^, and SO_4_^2−^), and the results are summarized in Table S5. As shown in Table S5, the K_d_ values for all tested resins decrease with increasing concentrations of competing anions, indicating a reduction in adsorption selectivity under high ionic strength conditions. Among the resins, PS-QA exhibits the highest K_d_ values in most cases, particularly at low competing anion concentrations, which reflects its superior selectivity toward the target adsorbate.

### 3.5. Regeneration Stability and Pilot-Scale Application in Fireworks Wastewater Treatment

**Figure 6:**
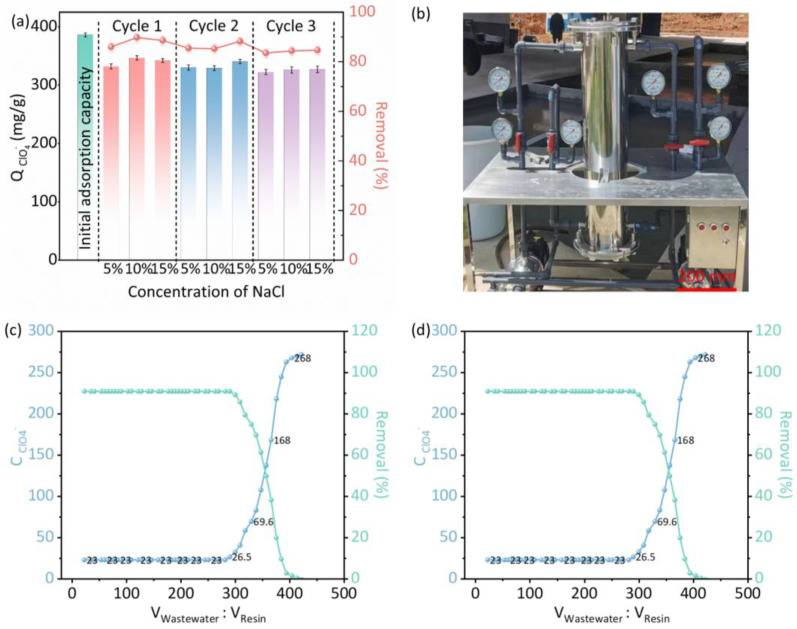
(**a**) Perchlorate removal efficiency of PS-QA resins during repeated use cycles. (**b**) Pilot-scale adsorption column used for fireworks wastewater treatment. Effect of influent flow rate on perchlorate removal by PS-QA resin in fireworks enterprise wastewater: (**c**) 5 BV, (**d**) 10 BV.

The reusability of PS-QA was evaluated using NaCl solutions of varying concentrations as regenerants (Figure 6a). After three adsorption–desorption cycles, ClO_4_^−^ removal efficiencies remained above 80%, despite slight declines. Among the regenerants, the 15% NaCl solution yielded the highest regeneration efficiency; however, the differences relative to the 5% and 10% NaCl solutions were marginal. Considering economic feasibility and resource conservation, we selected a 5% NaCl solution as the preferred regenerant for practical applications. Overall, the high stability and reusability of PS-QA underscore its strong potential for large-scale ClO_4_^−^ removal from complex fireworks wastewater.

Pilot-scale experiments were subsequently conducted using actual fireworks wastewater, employing a treatment train consisting of turbidity removal, aeration pretreatment, and resin-based deep adsorption. The properties of real wastewater are presented in Table S3, where the concentrations of ClO_4_^−^, Cl^−^, SO_4_^2−^, and NO_3_^−^ are 267, 28.3, 57.3, and 74.6 mg/L, respectively. Turbidity removal was achieved using hollow-fiber ultrafiltration membranes to reduce suspended solids, while ozonation-based aeration pretreatment decreased organic loading and prevented odor formation, thereby markedly improving wastewater clarity (Figure S6). PS-QA was applied in the third stage as a selective ClO_4_^−^ adsorbent and packed into a fixed-bed adsorption column. In this pilot-scale experiment, the bed volume was 50 L, the empty bed contact time (EBCT) was 6 min, and the regeneration was performed via counter-current elution with 10% NaCl solution. The column height was 800 mm and could be loaded with 10–25 L of resin (Figure 6b), allowing flexibility to accommodate variations in ClO_4_^−^ concentrations among different facilities. The influent wastewater contained 267 mg/L ClO_4_^−^, which is approximately 800 times higher than the maximum concentration permitted by the drinking water standard. Experiments conducted at flow rates of 5 and 10 BV/h (Figure 6c,d) showed slightly earlier breakthrough at the higher flow rate; nevertheless, the system sustained continuous operation for more than 16 h/day, producing 5 m^3^/day of effluent with ClO_4_^−^ concentrations near the detection limit and removal efficiencies exceeding 91%.

## 4. Conclusions

PS-QA, a novel quaternary ammonium-modified resin, was synthesized by functionalizing PVBC with *N*,*N*-dimethylethanolamine and demonstrated high selectivity and efficiency for ClO_4_^−^ removal. This macroporous resin exhibited a specific surface area of 19.94 m^2^/g, an average pore diameter of 32 nm, and a pore volume of 0.157 cm^3^/g, leading to adsorption performance superior to that of commercial resins. PS-QA achieved adsorption equilibrium within 60 min, and ClO_4_^−^ uptake conformed to the Freundlich isotherm model, indicating the coexistence of physical and chemical adsorption mechanisms. Optimal adsorption performance was observed under neutral conditions, and the resin exhibited strong resistance to interference in binary systems. Moreover, PS-QA demonstrated excellent reusability, retaining more than 80% of its active adsorption sites after three regeneration cycles, and showed outstanding performance in pilot-scale treatment of actual fireworks wastewater, highlighting its strong potential for practical applications.

## Figures and Tables

**Table 1:** Physicochemical properties of tested resins.

Resin	Skeleton	Functional Groups	Pore Volume (cm^3^/g)	Specific Surface Area (m^2^/g)	Average Pore Size (nm)	Ion-Exchange Capacity (mmol/g)
PS-QA	Polystyrene	–[R-N^+^(CH_3_)_2_(C_2_H_4_OH)]Cl^−^	0.157	19.94	32	3.7
PW521	Acrylics	–[R-N^+^(CH_3_)_3_]Cl^−^	0.046	6.94	28	3.1
BDX01	Polystyrene	–[R-N^+^(CH_3_)_3_]Cl^−^	0.155	19.7	29	3.3
BAX01	Polystyrene	–[R-N^+^(CH_2_CH_2_CH_3_)_3_]Cl^−^	0.010	5.01	15	3.0

## Data Availability

The original contributions presented in this study are included in the article/Supplementary Materials. Further inquiries can be directed to the corresponding author.

## Supplementary Materials

The following supporting information can be downloaded at https://www.mdpi.com/article/10.3390/polym18050553/s1, Text S1: Preparation of anion-exchange resins; Text S2: Adsorption kinetics model; Text S3: Adsorption isotherm model; Figure S1: Synthesis route of quaternary ammonium resins; Figure S2: Appearance of the resins; Figure S3: Surface electrostatic potential in (a) PW521 and (b) BAX01; Figure S4: The influence of pH on ClO4^−^ absorption; Figure S5: The Zeta potential of PS-QA; Figure S6: The fireworks wastewater. From left to right are raw water, membrane filtrate, and water after deep resin treatment; Table S1: Kinetic parameters for perchlorate adsorption by tested resins at different temperatures; Table S2: Fitting isotherm parameters of perchlorate uptake by the tested resins at different temperatures; Table S3: Chemical parameters of fireworks production wastewater; Table S4: Comparison of the adsorption performance with reported resins; Table S5: The distribution coefficients (Kd) of the tested resins.
