# Supplementary material for: A Quaternary Ammonium-Modified Resin for Selective Perchlorate Removal from Fireworks Wastewater
Source: Polymers (Basel). 2026 Feb 25;18(5):553. doi: 10.3390/polym18050553 (PMC12986638; doi:10.3390/polym18050553)
Supplement: Supplementary file 1 [file polymers-18-00553-s001.zip › polymers-4113139-supplementary.pdf]

Supporting Information

A Quaternary Ammonium-Modified Resin for Selective Perchlorate Removal from Fireworks Wastewater

Fei He [1], Jiacheng Li [1], Zhipeng Pei [2], Yuhao Zhao [2] and Yiping Li [1,*]

[1] State Key Laboratory of Water Cycle and Water Security in River Basin, College of Environment, Hohai University, Nanjing 210098, China

[2] Nanjing Institute of Environmental Sciences, Ministry of Ecology and Environment, Nanjing, 210042, China

* Correspondence: liyiping@hhu.edu.cn

Text S1.Preparation of anion-exchange resins

Supplementary Text S1: The polystyrene anion exchange resins were synthesized through the amination of chloromethyl polystyrene beads. Initially, 10 g of poly vinylbenzyl chloride (PVBC) precursor resins were swollen and stirred in 80 ml of dioxane at room temperature in a three-necked flask for 12 h, and then reacted with N,N-Dimethylethanolamine) Molar ratio of N,N-dimethylethanolamine to PVBC: 1.5:1. PVBC with a chloromethyl substitution degree of 4.2 mmol/g was used as the precursor. The mixture solution was composed of 80 ml, amination agent, 80 mL dioxane, and was shifted to 250 ml, three-necked round bottle flask with gentle stirring for reflux condensation. The reaction was carried out at 80 °C for 24 h under an inert nitrogen atmosphere to avoid oxidation and side reactions, the resin beads were rinsed with 1 M NaOH and 1 M HCl for two times, and then ethanol and distilled water alternately to neutral pH. The product was further purified by Soxhlet extraction with ethanol for 24 h to ensure the complete removal of unreacted amines and organic byproducts. Finally, the resin was dried under vacuum at 60 °C until a constant weight was obtained.

Text S2. Adsorption kinetics model

To further investigate the adsorption kinetics of different resins toward perchlorate, the pseudo-first-order, the pseudo-second-order and the intra-particle diffusion models were used to fit the adsorption kinetic data according to the following equations:

Pseudo-first-order model: $Q_t = Q_e(1 - \exp(-k_1 t))$

Pseudo-second-order model: $Q_t = (k_2 t Q_e^2)/(1 + k_2 t Q_e)$

Intra-particle diffusion model: $Q_t = k_i(t)^{1/2} + C$

where Q_e (mg/g) and Q_t (mg/g) denote the equilibrium adsorption capacity and adsorption capacity at time *t*, respectively; k_1 (min^{-1}) and k_2 (g/(mg min)) are the constants of pseudo-first-order and pseudo-second-order equations, respectively. The k_i (mmol g^{-1} min$^{1/2}$) represents the intraparticle diffusion rate and C is the intercept of the plot.

Text S3.Adsorption isotherm model

Langmuir model: $Q_e = Q_m K_L C_e/(1 + K_L C_e)$

Freundlich model: $Q_e = K_F C_e^{1/n}$

where Q_e (mg/g) and C_e (mg/L) are the solid-phase concentration and equilibrium aqueous-phase concentration, respectively. K_L and $K_F$represent the Langmuir and Freundlich capacity coefficient, respectively. Q_m (mg/g) is the maximum adsorption capacity. *n* is the Freundlich model parameter, which can reflect the adsorption intensity.

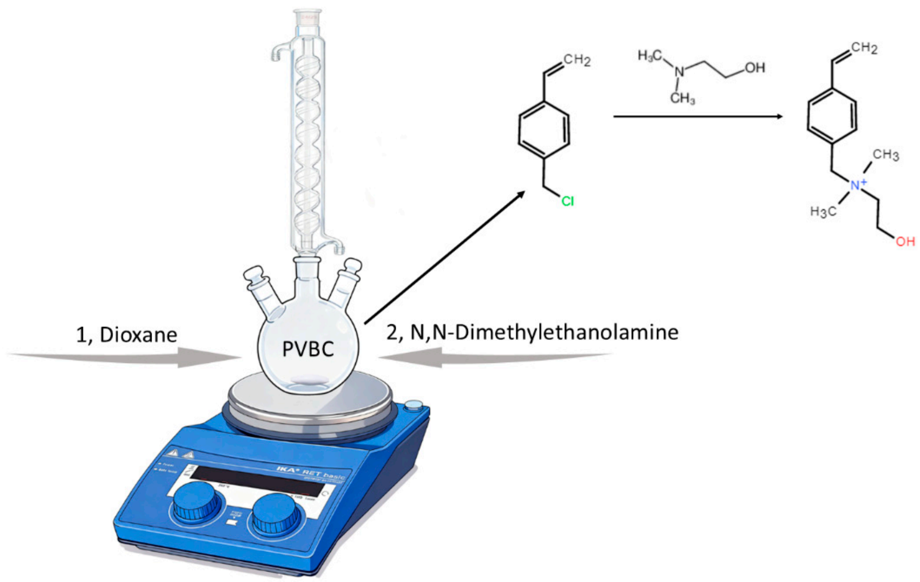

Figure S1. Synthesis route of quaternary ammonium resins.

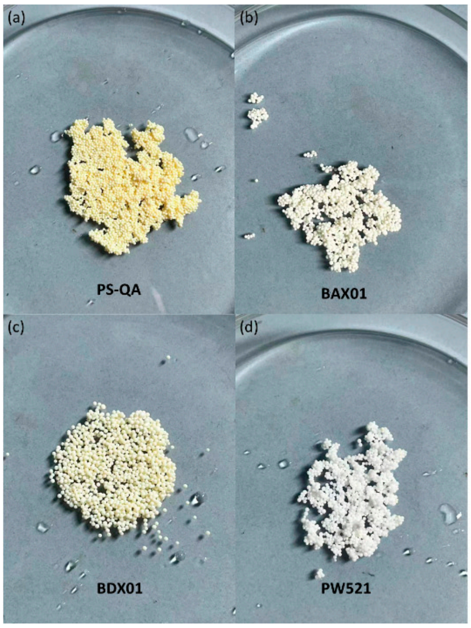

Figure S2. Appearance of the resins.

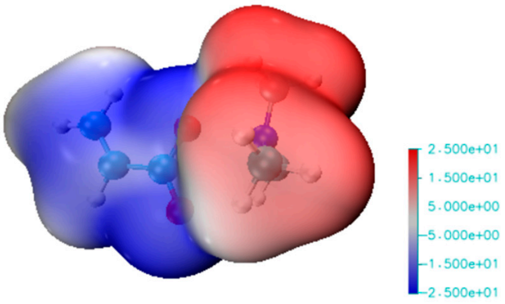

(a)

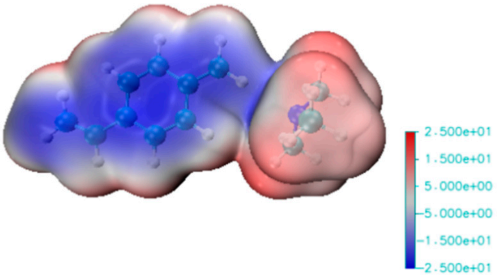

(b)

Figure S3. Surface electrostatic potential in (a) PW521 and (b) BAX01.

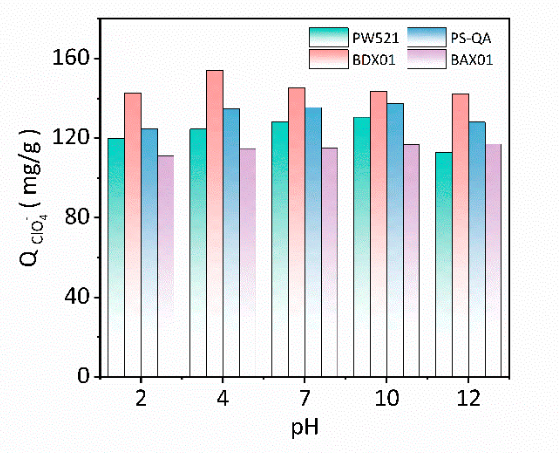

Figure S4. The influence of pH on ClO_4^- absorption.

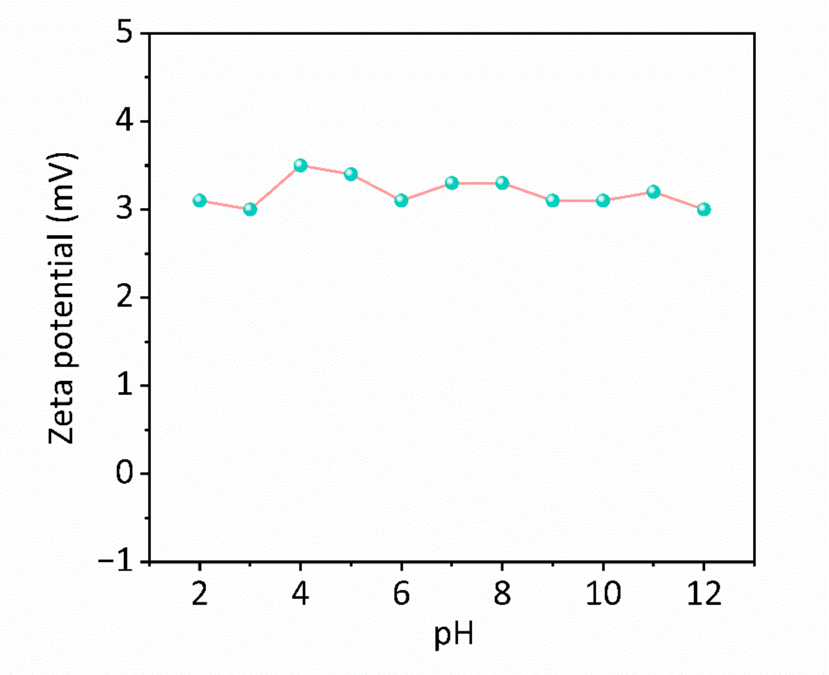

Figure S5. The Zeta potential of PS-QA.

The Zeta potential values stay positive at pH 2, 4, 7, 10, and 12 (no charge reversal to negative values).This ensures the material surface retains sufficient positive charge to electrostatically attract and adsorb the anionic ClO_4^-, even at high pH.

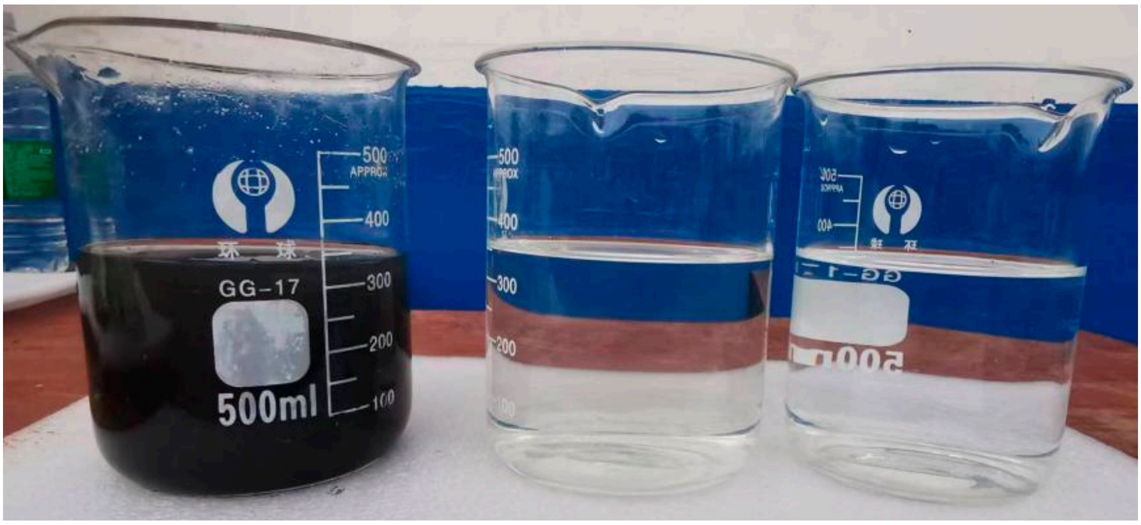

Figure S6. The fireworks wastewater. From left to right are raw water, membrane filtrate, and water after deep resin treatment.

Table S1. Kinetic parameters for perchlorate adsorption by tested resins at different temperatures.

Resin	Temperature （K）	Pseudo-first-order model			Pseudo-second-order model		
		K_1	Q_e	R^2	K_2	Q_e	R^2
PS-QA	295	0.0817	175.8	0.992	0.00111	178.2	0.994
	305	0.0965	173.8	0.993	0.00164	174.4	0.994
	315	0.1055	171.9	0.996	0.00184	173.3	0.997
BDX01	295	0.0800	184.5	0.984	0.00089	190.5	0.999
	305	0.0860	184.3	0.985	0.00101	189.7	0.999
	315	0.0958	183.0	0.991	0.00131	186.1	0.998
BAX01	295	0.0863	165.5	0.993	0.00123	169.0	0.995
	305	0.0919	165.8	0.995	0.00146	167.2	0.995
	315	0.0965	165.3	0.997	0.00180	165.7	0.993
PW521	295	0.1567	154.6	0.998	0.00597	154.8	0.997
	305	0.1520	156.5	0.996	0.00457	156.7	0.998
	315	0.1780	154.0	0.999	0.00596	155.0	0.999

Table S2. Fitting isotherm parameters of perchlorate uptake by the tested resins at different temperatures.

Resin	Temperature (K)	Langmuir			Freundlich		
		Q_m	K_L	R^2	1/n	K_F	R^2
**PS-QA**	295	371.3	0.30	0.922	0.271	116.3	0.962
	305	348.9	0.41	0.934	0.234	125.0	0.950
	315	339.7	0.48	0.953	0.230	123.8	0.943
**BDX01**	295	334.1	0.15	0.866	0.245	101.6	0.913
	305	307.3	0.21	0.879	0.255	92.7	0.972
	315	322.2	0.09	0.843	0.277	80.0	0.996
**BAX01**	295	293.5	0.03	0.486	0.259	62.0	0.869
	305	311.6	0.02	0.466	0.258	62.8	0.864
	315	300.8	0.024	0.490	0.260	62.3	0.872
**PW521**	295	197.3	0.28	0.623	0.202	70.4	0.843
	305	203.5	0.30	0.604	0.209	71.1	0.848
	315	210.3	0.26	0.670	0.210	72.6	0.872

Table S3. Chemical parameters of fireworks production wastewater.

Parameter	Units	Value
pH	-	6.8~8.0
Perchlorate (ClO_4^-)	mg/L	267
Chloride (Cl^-)	mg/L	28.3
Sulfate (SO_4^{2-})	mg/L	57.3
Nitrate (NO_3^-)	mg/L	74.6

Table S4. comparison of the adsorption performance with reported resins.

Resin	Capacity (g/L)	Selectivity	pH	Regeneration Performance
**A530E**	40–50	Ultra-high	2–11	Renewable with NaCl/NaOH
**A532E**	30–40	High	2–11	Primary disposable,
**D891**	≥25	High	3–9	Renewable with NaCl/NaOH
**RTA-17**	≥25	High	3–9	Renewable with NaCl/NaOH
**PS-QA**	≥60	Ultra-high	2-12	Renewable with 10% NaCl

Table S5. The distribution coefficients (K_d) of the tested resins.

	PW521	BDX01	BAX01	PS-QA
The concentration of NO_3^-				
0.5 mM	270.2528	1306.961	11933.69	34622.22
2 mM	160.2575	11697.67	4191.845	32903.30
5 mM	86.56166	8006.971	2107.506	8164.463
10 mM	45.21768	2741.61	1523.377	3167.974
20 mM	16.51231	1271.946	1041.553	549.3506
The concentration of Cl^-				
0.5 mM	1361.988	26009.66	8595.652	39582.54
2 mM	640.7407	18181.54	29930.03	29573.59
5 mM	455.5556	10629.61	3998.361	18947.62
10 mM	320.1681	6942.254	12322.36	10864.91
20 mM	231.1258	3645.318	2647.253	6393.506
The concentration of SO_4^{2-}				
0.5 mM	549.3506	27372.53	23429.41	32158.06
2 mM	235.5705	10338.41	7836.508	17141.38
5 mM	174.7253	7146.377	4229.004	2948.78
10 mM	93.05019	7900	2375.248	8520.69
20 mM	135.8491	6478.947	1949.18	6229.114
